# Masks, money, and mandates: A national survey on efforts to increase COVID-19 vaccination intentions in the United States

**DOI:** 10.1371/journal.pone.0267154

**Published:** 2022-04-21

**Authors:** Rikki H. Sargent, Shaelyn Laurie, Leah Moncada, Leo F. Weakland, James V. Lavery, Daniel A. Salmon, Walter A. Orenstein, Robert F. Breiman

**Affiliations:** 1 RIWI Corp., Toronto, Ontario, Canada; 2 Center for Global Health Innovation, Atlanta, Georgia, United States of America; 3 Hubert Department of Global Health, Rollins School of Public Health, Emory University, Atlanta, Georgia, United States of America; 4 Center for Ethics, Emory University, Atlanta, Georgia, United States of America; 5 Institute for Vaccine Safety, Johns Hopkins Bloomberg School of Public Health, Baltimore, Maryland, United States of America; 6 Department of International Health, Johns Hopkins Bloomberg School of Public Health, Baltimore, Maryland, United States of America; 7 Department of Health, Behavior and Society, Johns Hopkins Bloomberg School of Public Health, Baltimore, Maryland, United States of America; 8 School of Medicine, Emory University, Atlanta, Georgia, United States of America; Texas Tech University Health Science, Lubbock, UNITED STATES

## Abstract

Various efforts to increase COVID-19 vaccination rates have been employed in the United States. We sought to rapidly investigate public reactions to these efforts to increase vaccination, including self-reported responses to widespread reduced masking behavior, monetary incentive programs to get vaccinated, and work vaccination requirements. Using a unique method for data collection (Random Domain Intercept Technology), we captured a large (*N* = 14,152), broad-based sample of the United States Web-using population (data collected from June 30 –July 26, 2021). About 3/4 of respondents reported being vaccinated. The likelihood of vaccination and vaccination intention differed across various demographic indicators (e.g., gender, age, income, political leaning). We observed mixed reactions to efforts aimed at increasing vaccination rates among unvaccinated respondents. While some reported that specific efforts would increase their likelihood of getting vaccinated (between 16% and 32%), others reported that efforts would decrease their likelihood of getting vaccinated (between 17% and 42%). Reactions differed by general vaccination intention, as well as other demographic indicators (e.g., race, education). Our results highlight the need to fully understand reactions to policy changes, programs, and mandates before they are communicated to the public and employed. Moreover, the results emphasize the importance of understanding how reactions differ across groups, as this information can assist in targeting intervention efforts and minimizing potentially differential negative impact.

## Introduction

As of January 5, 2022, 85.9% of the United States adult population had received at least one COVID-19 vaccination [1]. Immunizing the remaining 36 million adults is a challenging and complicated task, as there are a variety of reasons that underpin vaccination decision-making. Although COVID-19 vaccinations are effective at preventing severe disease and death—even with high prevalence of infections due to SARS CoV2 variants (e.g., [2, 3])—national polls, such as those conducted by the Kaiser Family Foundation, have found that almost half of the unvaccinated population are firmly resistant to COVID-19 vaccination [4]. Some local governments have implemented monetary incentive programs to increase vaccinations [5]. Workplace vaccination mandates have also been imposed [6], and the removal of mask mandates for vaccinated citizens could encourage some people to seek vaccination. However, relaxed mask wearing requirements, monetary incentive programs, and work vaccination mandates could backfire, tightening resistance to vaccination within some demographic groups (e.g., [7, 8]). Understanding reactions to these measures (e.g., becoming more or less willing to get vaccinated) would be helpful in designing effective, data-driven policies, interventions, and programs.

Most previous studies on reactions to behavior change efforts implement an experimental design and/or aim to uncover complex theory-based processes (e.g., research on psychological reactance), and they often do not focus on identifying groups who may respond negatively (or positively) to efforts aimed at increasing intentions to get vaccinated. While this approach and aim are crucial to the expansion of scientific knowledge, the findings are often not directly and/or immediately relevant to policy makers, community leaders, intervention specialists, and business leadership. There is a need for direct assessment of reactions that can be immediately applied in a real-world setting. The current work was conducted by the CDC-funded COVID Vaccines Information Equity and Demand creation (COVIED) program, focused on evidence-based context-specific/tailored messaging to increase vaccination, especially among minority and hard-to-reach populations. In line with the program’s aim, this work emphasizes the need to rapidly assess public sentiment broadly and among subpopulations to characterize how different groups perceive and respond to COVID immunization campaigns. To do this, we harnessed a unique data collection method that allowed us to reach the broadest, most diverse population of respondents possible quickly, so that we could in turn inform policy as it was being formed/implemented.

We set out to assess reactions to widespread reduced masking behavior and efforts to increase COVID-19 vaccination rates (i.e., monetary incentive programs and work vaccination requirements). We aimed to characterize reactions among the unvaccinated population, as well as identify differences across general vaccination intention level and basic demographic characteristics (e.g., age, gender, race). We conducted this assessment using a unique method for data collection that allowed us to capture a large, broad-based sample of the Web-using population quickly across the United States—in doing so, we were able to rapidly reach subpopulations and uncover differential reactions that could shed light on issues surrounding vaccine equity and could, in turn, inform messaging and policy.

## Method

### Procedure and design

We conducted an internet-based cross-sectional survey collecting data from June 30 –July 26, 2021, using a standardized survey format. We utilized RIWI (Real-Time Interactive World-Wide Intelligence) technology to engage with a sample of the US Web-using population. RIWI’s Random Domain Intercept Technology works as follows: first, Web users land on an inactive (broken) Web domain that RIWI temporarily manages. RIWI then validates the Web user’s country location and delivers the appropriate survey to the Web user. Web users choose whether to safely and anonymously opt-in to participate in the study, and they may end their participation at any time. Unlike panel surveys, no incentives are provided and no personally identifiable information is exchanged. RIWI technology has been described as nonprobability, online intercept sampling [9], and has been previously employed to assess various topics such as health care quality [10–12], mental illness stigma [13–15], and anti-vaccine sentiment [16].

Participants first identified whether they would like to complete the survey in English or Spanish. Following the language selection, participants reported their age (to participate, respondents had to be 18 years of age or older), gender, and COVID-19 vaccination status. Those who reported receiving at least one dose of a COVID-19 vaccine answered several questions about the vaccine (e.g., which vaccine they received and when). Those who reported that they were unvaccinated were asked up to 17 questions (depending on skip/display logic) surrounding their vaccination intentions, reactions to reduced mask-wearing, incentive programs, and work vaccination requirements, reasons for not getting vaccinated, and discussions with healthcare providers about vaccination, among other vaccination-related questions. All respondents (regardless of vaccination status) completed measures assessing their primary daily news source, entertainment sources, and basic demographics (i.e., race, education, living location, political leaning, and annual household income). We designed the survey to take less than 5 minutes to complete. The Institutional Review Boards at Emory University and Johns Hopkins University designated this project as public health practice (not human subjects research). Respondents implied consent by voluntarily participating in the survey.

### Participants

Of the 1,026,850 Web-users who landed on the RIWI-operated inactive Web domain, 63,853 opted in, were eligible to participate (18+ years old, located in the US), and answered the first three questions of the survey (age, gender, vaccination status). Among those, 14,152 completed the entirety of the survey (22%) and were included in the current analysis (see S1 Fig for survey progression). Participant demographics are displayed in Table 1.

**Table 1:** Demographic characteristics of the full sample, vaccinated subsample, and unvaccinated subsample.

	Total (*N* = 14,152)		Vaccinated (*N* = 10,866)		Unvaccinated (*N* = 3,286)	
**Age**						
18–25	2,702	(19.1%)	1,967	(18.1%)	735	(22.4%)
26–35	2,280	(16.1%)	1,588	(14.6%)	692	(21.1%)
36–45	2,149	(15.2%)	1,540	(14.2%)	609	(18.5%)
46–55	2,103	(14.9%)	1,617	(14.9%)	486	(14.8%)
56–64	1,803	(12.7%)	1,466	(13.5%)	337	(10.3%)
65–74	1,759	(12.4%)	1,559	(14.3%)	200	(6.1%)
75–84	725	(5.1%)	658	(6.1%)	67	(2.0%)
85+	631	(4.5%)	471	(4.3%)	160	(4.9%)
**Gender**						
Male	7,097	(50.1%)	5,537	(51.0%)	1,560	(47.5%)
Female	6,305	(44.6%)	4,804	(44.2%)	1,501	(45.7%)
Other	750	(5.3%)	525	(4.8%)	225	(6.8%)
**Race**						
White	8,151	(57.6%)	6,352	(58.5%)	1,799	(54.7%)
Black	1,656	(11.7%)	1,210	(11.1%)	446	(13.6%)
Latinx	1,476	(10.4%)	1,119	(10.3%)	357	(10.9%)
Asian	793	(5.6%)	696	(6.4%)	97	(3.0%)
NA/AN	340	(2.4%)	244	(2.2%)	96	(2.9%)
Other	706	(5.0%)	456	(4.2%)	250	(7.6%)
Multi-racial	1,030	(7.3%)	789	(7.3%)	241	(7.3%)
**Education**						
High school or less	4,498	(31.8%)	3,140	(28.9%)	1,358	(41.3%)
Tech/vocational training	1,915	(13.5%)	1,397	(12.9%)	518	(15.8%)
College degree	4,803	(33.9%)	3,852	(35.5%)	951	(28.9%)
Masters or higher	2,936	(20.7%)	2,477	(22.8%)	459	(14.0%)
**Living Location**						
Large city	3,969	(28.0%)	3,096	(28.5%)	873	(26.6%)
Suburb	4,607	(32.6%)	3,709	(34.1%)	898	(27.3%)
Town/village	3,172	(22.4%)	2,373	(21.8%)	799	(24.3%)
Rural area/farm	2,404	(17.0%)	1,688	(15.5%)	716	(21.8%)
**Political Leaning**						
Democrat	4,015	(28.4%)	3,466	(31.9%)	549	(16.7%)
Independent, lean Democrat	1,663	(11.8%)	1,401	(12.9%)	262	(8.0%)
Independent	3,985	(28.2%)	2,809	(25.9%)	1,176	(35.8%)
Independent, lean Republican	1,410	(10.0%)	975	(9.0%)	435	(13.2%)
Republican	3,079	(21.8%)	2,215	(20.4%)	864	(26.3%)
**Income**						
Under $20,000	3,360	(23.7%)	2,377	(21.9%)	983	(29.9%)
$20,000 - $50,000	2,975	(21.0%)	2,218	(20.4%)	757	(23.0%)
$50,001 - $75,000	2,307	(16.3%)	1,770	(16.3%)	537	(16.3%)
$75,001 - $125,000	2,457	(17.4%)	2,016	(18.6%)	441	(13.4%)
$125,001 - $250,000	1,547	(10.9%)	1,321	(12.2%)	226	(6.9%)
Over $250,000	1,506	(10.6%)	1,164	(10.7%)	342	(10.4%)

NA/AN = Native American / Alaska Native. Income = annual household income.

### Primary measures

The primary measures reported in the current manuscript are described below. In addition to these items, we also measured demographic information on age, gender, race, education level, living location, political leaning, and annual household income. See S1 File for the full survey instrument.

#### Vaccination status and intention

We measured vaccination status and, among unvaccinated respondents, intentions to get vaccinated. The five response options for the vaccination intention item ranged from *I will definitely get it as soon as I can* to *I will definitely not get vaccinated*. For the current manuscript, we categorized respondents as either vaccinated (with at least one dose of a COVID-19 vaccine received) or unvaccinated, and, among those unvaccinated, as either leaning toward vaccination (definitely/likely to get vaccinated or likely to get vaccinated but not right away) or resistant to vaccination (definitely/likely to not get vaccinated).

#### Masking

We asked unvaccinated respondents how fewer people wearing masks impacted their intentions to get vaccinated (*more likely; less likely; no effect)*.

#### Monetary incentives

We asked unvaccinated respondents to indicate how monetary incentive programs ($25 gift card and $100,000 lottery) impacted their intention to get vaccinated (*I would be more likely to get vaccinated right away; I would be less likely to seek vaccination; there would be no effect on my getting vaccinated*).

#### Work requirements

We asked working unvaccinated respondents to indicate how employer vaccination requirements impacted their vaccination intentions (*I would get vaccinated; I would not get vaccinated; I am not sure what I would do*). Workers who said that they would not get vaccinated then reported what they would do instead (*quit my job; protest; consider legal action; other*).

### Analysis

We report on a series of chi-square tests, multiple logistic regression analyses, multinomial logistic regression analyses, and correlational analyses. We provide odds ratios in-text where appropriate, as well as the 95% confidence intervals surrounding any odds ratios that are not provided in the tables.

We first outline basic demographic differences in vaccination status (Table 1). We then further assess *unique* demographic predictors of vaccination status (vaccinated vs. unvaccinated) and vaccination intention among those unvaccinated (leaning toward vaccination vs. resistant to vaccination) using two multiple logistic regression models (Table 2). The models predict each variable as a function of the following factor variables: age group, gender, race, education, living location, political leaning, and annual household income. We next report results from a series of chi-square tests assessing the relationship between reactions to each effort and vaccination intention among those unvaccinated (Table 3).

**Table 2:** Multiple logistic regression analyses predicting vaccination status and intention (dichotomized) by demographics.

	Vaccinated (vs. Unvaccinated)			Leaning Toward (vs. Resistant)		
	*AOR*	*95% CI*	*p*	*AOR*	*95% CI*	*p*
**(Intercept)**	4.37	3.70 – 5.16	< .001	1.56	1.15 – 2.11	.004
**Age (ref = 18–25)**						
26–35	0.77	0.68 – 0.88	< .001	0.80	0.64 – 0.99	.045
36–45	0.84	0.74 – 0.96	.013	0.78	0.62 – 0.98	.032
46–55	1.10	0.96 – 1.27	.179	0.66	0.52 – 0.85	.001
56–64	1.51	1.30 – 1.77	< .001	0.69	0.52 – 0.92	.011
65–74	2.64	2.21 – 3.16	< .001	0.54	0.37 – 0.78	.001
75–84	3.32	2.54 – 4.41	< .001	0.56	0.31 – 0.99	.051
85+	1.21	0.97 – 1.51	.097	0.41	0.25 – 0.66	< .001
**Gender (ref = Male)**						
Female	0.82	0.75 – 0.89	< .001	0.88	0.76 – 1.03	.114
Other	0.69	0.57 – 0.85	< .001	0.65	0.44 – 0.96	.034
**Race (ref = White)**						
Black	0.78	0.68 – 0.89	< .001	1.37	1.09 – 1.72	.007
Latinx	1.13	0.98 – 1.29	.096	2.47	1.93 – 3.16	< .001
Asian	2.27	1.82 – 2.86	< .001	1.86	1.21 – 2.88	.005
NA/AN	0.84	0.65 – 1.09	.187	0.82	0.49 – 1.32	.430
Other	0.62	0.52 – 0.74	< .001	1.00	0.72 – 1.38	.988
Multi-racial	1.14	0.97 – 1.34	.122	1.30	0.97 – 1.73	.073
**Education (ref = High school or less)**						
Tech/vocational training	1.05	0.93 – 1.20	.426	1.04	0.83 – 1.30	.709
College degree	1.52	1.37 – 1.69	< .001	1.08	0.89 – 1.30	.452
Masters or higher	1.89	1.64 – 2.17	< .001	0.72	0.55 – 0.95	.022
**Living Location (ref = Large city)**						
Suburb	1.13	1.01 – 1.26	.033	1.05	0.86 – 1.29	.617
Town/village	0.93	0.83 – 1.05	.237	0.99	0.81 – 1.23	.949
Rural area/farm	0.80	0.71 – 0.91	< .001	0.75	0.60 – 0.94	.014
**Political Leaning (ref = Democrat)**						
Independent, lean Democrat	0.81	0.69 – 0.96	.012	0.97	0.71 – 1.32	.824
Independent	0.42	0.37 – 0.47	< .001	0.64	0.52 – 0.80	< .001
Independent, lean Republican	0.31	0.26 – 0.36	< .001	0.48	0.36 – 0.63	< .001
Republican	0.37	0.33 – 0.42	< .001	0.41	0.32 – 0.52	< .001
**Income (ref = Under $20,000)**						
$20,000 - $50,000	1.04	0.92 – 1.17	.523	0.79	0.64 – 0.96	.021
$50,001 - $75,000	1.05	0.92 – 1.20	.486	0.72	0.56 – 0.91	.006
$75,001 - $125,000	1.38	1.20 – 1.59	< .001	0.78	0.60 – 1.01	.062
$125,001 - $250,000	1.64	1.38 – 1.96	< .001	0.76	0.54 – 1.07	.116
Over $250,000	1.05	0.90 – 1.24	.534	0.69	0.51 – 0.94	.021

AOR = adjusted odds ratio. Ref = reference category. Income = annual household income. The AORs compare the given subgroup to the referent group, adjusting for all other variables in the model. For example, Asian respondents were 2.27 times more likely to be vaccinated than White respondents, adjusting for age, gender, education, living location, political leaning, and income. Model *R*^*2*^ Nagelkerke were 0.13 and 0.08 for the vaccination status and vaccination intention models, respectively.

**Table 3:** Chi-square analyses predicting impact on vaccination intention by general vaccination intention (dichotomized).

	Resistant	Leaning Toward	*X* ^*2*^	*df*	*p*
**Reduced Masking**					
More likely	106 (5.2%)	580 (45.8%)	786.69	2	< .001
No effect	1,544 (76.4%)	507 (40.0%)			
Less likely	370 (18.3%)	179 (14.1%)			
**Gift Card**					
More likely	161 (8.0%)	378 (29.9%)	387.12	2	< .001
No effect	1,047 (51.8%)	695 (54.9%)			
Less likely	812 (40.2%)	193 (15.2%)			
**Lottery**					
More likely	206 (10.2%)	438 (34.6%)	378.73	2	< .001
No effect	1,042 (51.6%)	637 (50.3%)			
Less likely	772 (38.2%)	191 (15.1%)			
**Work Requirement**					
Would vaccinate	146 (11.8%)	539 (60.3%)	728.10	2	< .001
Unsure of response	297 (23.9%)	252 (28.2%)			
Would not vaccinate	798 (64.3%)	103 (11.5%)			

To assess *unique* demographic differences in how reduced mask-wearing, monetary incentive programs, and work vaccination requirements impact intentions to get vaccinated, we conducted a series of multinomial logistic regression models predicting each outcome of interest as a function of the following factor variables: age group, gender, race, education, living location, political leaning, and annual household income. Due to the consistent observation of differences across general intention to vaccinate, we also adjusted for this individual difference in analyses. Of note, we originally aimed to perform a series of ordinal logistic regressions; however, our data violated the proportional odds assumption. As such, we performed multinomial logistic regressions and chose to highlight demographic comparisons between those who report being more likely to get vaccinated vs. those who report being less likely to get vaccinated in response to each vaccination effort (Table 4).

**Table 4:** Multinomial logistic regression analyses predicting impact on vaccination intention by demographics (more likely vs. less likely to get vaccinated comparison).

	Reduced Masking			Gift Card			Lottery			Work Requirement		
	*AOR*	95% CI	*p*	*AOR*	95% CI	*p*	*AOR*	95% CI	*p*	*AOR*	95% CI	*p*
**(Intercept)**	**0.37**	0.22 – 0.63	< .001	1.00	0.63 – 1.60	.992	1.20	0.76– 1.88	0.433	0.68	0.39 – 1.17	.160
**Intention (ref = Resistant)**												
Lean Toward	**10.46**	7.88 – 13.87	< .001	**9.02**	6.97 – 11.67	< .001	**7.47**	5.86 – 9.51	< .001	**26.99**	20.10 – 36.23	< .001
**Age (ref = 18–25)**												
26–35	1.12	0.78 – 1.61	.528	0.72	0.52 – 1.01	.061	0.74	0.53 – 1.02	.064	1.17	0.81 – 1.68	.404
36–45	**1.52**	1.04 – 2.22	.030	0.82	0.57 – 1.17	.271	**0.70**	0.50 – 0.98	.038	0.93	0.63 – 1.37	.704
46–55	1.29	0.86 – 1.95	.220	1.00	0.68 – 1.49	.992	0.76	0.52 – 1.10	.141	0.93	0.61 – 1.42	.748
56–64	**1.74**	1.08 – 2.79	.023	0.91	0.56 – 1.47	.687	0.85	0.54 – 1.33	.477	0.84	0.51 – 1.39	.507
65–74	**1.87**	1.01 – 3.48	.046	0.60	0.31 – 1.16	.126	**0.46**	0.24 – 0.87	.017	0.74	0.34 – 1.62	.450
75–84	1.07	0.44 – 2.65	.876	0.51	0.20 – 1.28	.151	**0.33**	0.12 – 0.88	.026	**4.20**	1.10 – 16.10	.036
85+	1.20	0.56 – 2.55	.643	1.12	0.62 – 2.03	.702	0.74	0.42 – 1.32	.314	0.59	0.22 – 1.63	.314
**Gender (ref = Male)**												
Female	1.06	0.82 – 1.37	.649	0.82	0.64 – 1.05	.110	0.87	0.69 – 1.10	.250	1.14	0.88 – 1.49	.324
Other	1.36	0.72 – 2.55	.341	1.67	0.99 – 2.82	.055	**1.78**	1.07 – 2.96	.027	0.72	0.33 – 1.58	.410
**Race (ref = White)**												
Black	1.07	0.74 – 1.54	.721	**0.48**	0.33 – 0.69	< .001	**0.61**	0.44 – 0.86	.005	0.73	0.49 – 1.08	.115
Latinx	1.17	0.79 – 1.72	.428	0.69	0.47 – 1.02	.063	0.77	0.53 – 1.11	.161	0.72	0.47 – 1.10	.133
Asian	1.18	0.64 – 2.17	.599	1.16	0.62 – 2.18	.647	0.72	0.39 – 1.33	.292	0.84	0.35 – 2.04	.705
NA/AN	1.01	0.47 – 2.19	.970	0.72	0.37 – 1.38	.319	**0.33**	0.16 – 0.71	.004	**0.16**	0.05 – 0.50	.001
Other	0.92	0.53 – 1.58	.764	**0.47**	0.29 – 0.75	< .001	**0.51**	0.33 – 0.80	.004	0.58	0.31 – 1.10	.095
Multi-racial	1.34	0.82 – 2.18	.243	**0.55**	0.35 – 0.88	.012	0.83	0.54 – 1.27	.395	1.04	0.62 – 1.73	.891
**Education (ref = High school or less)**												
Tech/vocational training	0.82	0.57 – 1.19	.301	1.16	0.81 – 1.65	.419	**1.45**	1.03 – 2.02	.032	**0.46**	0.31 – 0.68	< .001
College degree	0.82	0.60 – 1.12	.207	0.99	0.74 – 1.34	.968	0.97	0.73 – 1.30	.854	**0.62**	0.45 – 0.85	.003
Masters or higher	**0.60**	0.38 – 0.95	.029	0.67	0.44 – 1.02	.062	0.72	0.49 – 1.08	.109	**0.58**	0.36 – 0.94	.027
**Living Location (ref = Large city)**												
Suburb	0.95	0.68 – 1.33	.787	0.85	0.62 – 1.16	.308	0.79	0.59 – 1.07	.130	0.88	0.62 – 1.25	.466
Town/village	0.95	0.67 – 1.33	.756	**0.70**	0.51 – 0.97	.034	**0.63**	0.46 – 0.85	.003	0.84	0.59 – 1.20	.332
Rural area/farm	0.74	0.50 – 1.08	.119	**0.58**	0.41 – 0.83	.003	**0.68**	0.49 – 0.94	.020	**0.66**	0.44 – 0.98	.037
**Political Leaning (ref = Democrat)**												
Independent, lean Democrat	1.12	0.67 – 1.88	.670	0.74	0.45 – 1.19	.213	0.88	0.55 – 1.43	.616	1.01	0.57 – 1.76	.985
Independent	0.71	0.50 – 1.00	.053	**0.44**	0.32 – 0.61	< .001	**0.55**	0.41 – 0.75	< .001	**0.62**	0.42 – 0.91	.015
Independent, lean Republican	**0.49**	0.30 – 0.79	.004	**0.25**	0.15 – 0.39	< .001	**0.24**	0.16 – 0.38	< .001	**0.34**	0.20 – 0.57	< .001
Republican	**0.56**	0.37 – 0.84	.005	**0.25**	0.17 – 0.37	< .001	**0.30**	0.21 – 0.43	< .001	**0.54**	0.35 – 0.83	.005
**Income (ref = Under $20,000)**												
$20,000 - $50,000	1.17	0.84 – 1.63	.344	1.31	0.95 – 1.80	.096	1.13	0.83 – 1.54	.426	0.99	0.70 – 1.40	.945
$50,001 - $75,000	1.07	0.73 – 1.58	.723	0.83	0.57 – 1.22	.352	1.09	0.76 – 1.55	.648	0.83	0.55 – 1.24	.366
$75,001 - $125,000	0.83	0.53 – 1.30	.418	**0.56**	0.36 – 0.87	.009	0.76	0.51 – 1.13	.181	0.70	0.44 – 1.09	.114
$125,001 - $250,000	0.92	0.51 – 1.66	.789	0.73	0.42 – 1.28	.275	1.02	0.61 – 1.69	.950	**0.47**	0.26 – 0.83	.009
Over $250,000	0.96	0.58 – 1.59	.871	0.83	0.53 – 1.30	.413	0.70	0.46 – 1.08	.105	**0.45**	0.25 – 0.81	.008

*AOR* = adjusted odds ratio. Ref = reference category. Income = annual household income. The AORs compare the given subgroup to the referent group, adjusting for all other variables in the model. For example, those leaning toward vaccination were 9.02 times more likely to get vaccinated in response to a gift card incentive than those resistant to vaccination, adjusting for age, gender, race, education, living location, political leaning, and income. Model *R*^*2*^ Nagelkerke ranged from 0.23 to 0.41. Coefficients significant at *p* < .05 are in bold.

Finally, we performed correlational analyses to assess the relationships between reactions to reduced mask-wearing, monetary incentives, and work vaccination requirements.

## Results

### Vaccination status and intention

Of the 63,853 individuals who opted in to take the survey, 44,524 (69.7%) were vaccinated and 19,329 (30.3%) were unvaccinated (manuscript submitted for publication). The survey designed for unvaccinated respondents was longer than the survey designed for vaccinated respondents. As such, we retained proportionately more vaccinated respondents relative to unvaccinated respondents in the final analytic sample. Among the analytic sample (i.e., those who completed the entire survey), 10,866 (76.8%) had received at least one dose of a COVID-19 vaccine at the time of data collection and 3,286 (23.2%) respondents were unvaccinated.

We first assessed demographic indicators of vaccination status without adjusting for other demographic variables (Table 1). Those 46 years of age or older were 1.8 [1.7, 2.0] times more likely to be vaccinated than those 45 years of age or younger. Those with more than a high school education were 1.7 [1.6, 1.9] times more likely to be vaccinated than those with a high school education or less. Those living in towns/villages, suburbs, or large cities were 1.5 [1.4, 1.7] times more likely to be vaccinated than those living in rural/farm areas. Individuals who identified as Democrat or Democrat-leaning Independent were 2.5 [2.3, 2.7] times more likely to be vaccinated than those who identified as Independent, Republican-leaning Independent, or Republican. Those with annual household incomes greater than $50,001 were 1.5 [1.4, 1.7] times more likely to be vaccinated than those with annual household incomes at or under $50,000.

Among the unvaccinated respondents, 15.6% indicated that they would definitely get vaccinated as soon as they could, 5.7% that they would likely get vaccinated as soon as they could, 17.3% that they would likely get vaccinated but not right away, 18.7% that they would likely not get vaccinated, and 42.8% that they would definitely not get vaccinated (i.e., 38.5% were leaning toward vaccination and 61.5% were resistant).

#### Vaccination status

Adjusting for all demographic variables (Table 2), those ages 56+ were 2.1 [1.9, 2.3] times more likely to be vaccinated than those 55 years of age or younger. Males were 1.2 [1.1, 1.3] times more likely to be vaccinated than females. White respondents were 1.3 [1.1, 1.5] times more likely to be vaccinated than Black respondents, whereas Asian respondents were 2.3 times more likely to be vaccinated than White respondents. Those with college degrees or higher were 1.6 [1.5, 1.7] times more likely to be vaccinated than those with technical/vocational training or less. Those living in large cities or suburbs were 1.2 [1.1, 1.3] times more likely to be vaccinated than those living in towns/villages or rural/farm areas. Democrats and Democrat-leaning Independents were 2.4 [2.2, 2.7] times more likely than Independents, Republican-leaning Independents, and Republican respondents to be vaccinated. Those with annual household incomes between $75,001-$250,000 were 1.4 [1.3, 1.6] times more likely to be vaccinated than those with household incomes under $75,000.

#### Vaccination intention

Adjusting for all demographic variables (Table 2), those unvaccinated and from the youngest age group (18–25 years old) were 1.4 [1.2, 1.7] times more likely to lean toward vaccination relative to those unvaccinated and 26 years of age and older. Unvaccinated Black, Latinx, and Asian respondents were 1.4, 2.5, and 1.9 times more likely to lean toward vaccination relative to unvaccinated White respondents. Unvaccinated Democrats and Democrat-leaning Independents were 1.8 [1.5, 2.2] times more likely to lean toward vaccination, than Independents, Republican-leaning Independents, and Republican respondents.

### Masking

Among unvaccinated respondents, 20.9% reported that fewer people wearing masks in public made them more likely to get vaccinated, whereas 16.7% said it made them less likely to get vaccinated, and the majority (62.4%) said it had no effect on their vaccination intentions. These reactions differed by overall intention to get vaccinated (Table 3). Those leaning toward vaccination were 11.3 [8.6, 14.9] times more likely than those resistant to vaccination to report an increased (vs. decreased) likelihood of getting vaccinated in response to reduced mask-wearing.

### Monetary incentives

Among unvaccinated respondents, only 16.4% reported that a $25 gift card would make them more likely to get vaccinated, whereas 30.6% said it would make them less likely to get vaccinated and 53.0% said it would have no effect on their vaccination intention. Reactions to the $100,000 lottery incentive were similar– 19.6% said they would be more likely to get vaccinated, 29.3% said they would be less likely to get vaccinated, and 51.1% said the lottery incentive would have no impact on their vaccination intentions. These reactions again differed by overall intention (Table 3). Those leaning toward vaccination were 9.9 [7.8, 12.6] and 8.6 [6.8, 10.8] times more likely than the resistant population to report an increased (vs. decreased) likelihood of getting vaccinated in response to the gift card and lottery incentive, respectively.

### Work requirements

Among working unvaccinated respondents (*N* = 2,135), 32.1% said that they would get vaccinated in response to a work requirement, 42.2% said they would not get vaccinated, and 25.7% reported that they were unsure if they would get vaccinated. These reactions to work requirements differed by overall vaccination intention (Table 3). Those leaning toward vaccination were 28.6 [21.8, 37.8] times more likely than resistant unvaccinated workers to get vaccinated (vs. refuse vaccination) in response to a workplace requirement. Among those who reported that they would not get vaccinated despite a mandate (*n* = 901), 43.1% said that they would consider legal action, 30.1% would quit their jobs, 9.0% would protest, and 17.9% chose an unspecified course of action (see S1 Table for an analysis of demographic differences in anticipated reactionary responses).

### Demographic differences

Adjusting for all demographic indicators (Table 4), those leaning toward vaccination were 7.5 to 27.0 times more likely to report an increased (vs. decreased) likelihood of getting vaccinated in response to each vaccination effort (i.e., reduced masking, gift card incentive, lottery incentive, and workplace requirements). Those who identified as Democrat or Democrat-leaning Independent were 1.6 [1.2, 2.2] to 2.6 [2.0, 3.4] times more likely to report an increased (vs. decreased) likelihood of getting vaccinated in response to each effort relative to those who identified as Independent, Republican-leaning Independent, or Republican. Those with high school educations or less were 1.7 [1.1, 2.7] times more likely than those with masters degrees or higher to report an increased (vs. decreased) likelihood of getting vaccinated in response to reduced mask-wearing. White respondents were 2.1 [1.5, 3.0] and 1.6 [1.2, 2.3] times more likely than Black respondents to report an increased (vs. decreased) likelihood of getting vaccinated in response to a gift card and lottery incentive, respectively. Those living in large cities or suburbs were 1.4 [1.1, 1.8] and 1.4 [1.1, 1.7] times more likely to be vaccinated than those living in towns/villages or rural/farm areas in response to a gift card and lottery incentive, respectively. Those with high school educations or less were 1.8 [1.4, 2.4] times more likely than those with tech/vocational training or higher to report an increased (vs. decreased) likelihood of getting vaccinated in response to a workplace requirement. Finally, those with annual household incomes under $125,000 were 1.9 [1.3, 2.9] times more likely than those with incomes over $125,001 to report an increased (vs. decreased) likelihood of getting vaccinated in response to a workplace requirement.

### Relationships among reactions

Reactions were significantly and positively related to one another (at *p* < .001; see S2 Table for full correlation matrix). In general, people who respond positively to one program are likely to respond positively to others, and vice versa. However, most reactions were only moderately correlated with one another, indicating that there are nuances in reactions to different incentives/mandates that require further investigation.

## Discussion

At the time of data collection, about 3/4 of our respondents were vaccinated, and the likelihood of vaccination uniquely differed across all demographic indicators. For example, men were more likely to be vaccinated than women, those with lower education (technical/vocational training or less) were less likely to be vaccinated than those with higher education (college degree or higher), and Democrats were more likely to be vaccinated than those with other political leanings. We again saw unique demographic differences in vaccination intention among the unvaccinated respondent group (e.g., those in the youngest age group were more likely to lean toward vaccination than all other age groups). These findings support previous research identifying individual differences in vaccination rates and vaccination hesitancy based on race, income, and political leaning [17, 18], and they highlight the importance of continuing to monitor demographic differences in vaccination status/intention moving forward. Moreover, these findings emphasize the necessity for targeted assessment and messaging to meet the needs of different populations.

Reduced public masking (especially in response to changing public health guidelines) could differentially impact vaccination intentions. Although reduced masking made many of those already leaning toward vaccination more likely to get vaccinated, the positive impact is minimally observed among those resistant to vaccination. Perhaps more importantly though, we found that reduced mask-wearing deterred some individuals from seeking vaccination. While we did not collect data on this point, it is conceivable that reduced mask-wearing signaled to some people that the pandemic was winding down, so the need to “risk” immunization was correspondingly reduced. As we enter an endemic COVID-19 [19], society needs to be prepared for changing public health policies. However, policy makers should also be fully aware of how such changes might influence (and perhaps impede on) other efforts, such as efforts to increase vaccination.

Monetary incentive programs have been implemented across the United States, but it is unclear how unvaccinated individuals respond to these programs. We observed mixed reactions, with most unvaccinated respondents indicating that these programs have no impact on their intention, followed by large proportions (over a quarter) of respondents indicating that the programs make them less likely to get vaccinated. The promising effects of these programs on vaccination intention are more prevalent among those who are already leaning toward vaccination (vs. resistant to vaccination), but even so, less than half of respondents who are leaning toward vaccination say that the programs would make them more likely to get vaccinated. These findings support previous claims that monetary incentives do not have a widespread, positive impact on vaccination intention (e.g., [20]). Nonetheless, a recent program in Dekalb County, GA offering $100 for people receiving their first dose of a COVID-19 vaccine resulted in immunization of more than 2,500 people on a single Saturday in one location [21]. Clearly, there are nuances about incentive programs that need further characterization. Moreover, we should point out that, at the time of data collection, some of those who were recently vaccinated may have been favorably impacted by incentives and mandates, and thus were not included in our unvaccinated analyses.

As workplaces begin to require vaccinations, it is important to be prepared for potentially differential reactions among unvaccinated workers. We also observed mixed reactions to such requirements, with relatively equal proportions of respondents saying that they would get vaccinated, would not get vaccinated, and were unsure of if they would get vaccinated. These reactions again differed by intention, with a small proportion of those resistant to vaccination saying that they would get vaccinated in response to a work requirement. While such requirements might be helpful in pushing those leaning toward vaccination to get vaccinated, it is likely to be accompanied with widespread refusal and discontent among those already resistant to vaccination (which, we remind the reader, makes up a large proportion of the unvaccinated population). Such widespread negative reactance could result in legal action, protests, and loss of employees. Nonetheless, a substantial proportion of hospitals, major corporations, Universities, and the military are moving ahead with mandates (e.g., [6, 22]); it will be important to fully characterize the response to these incentives, including long term attitudes and behaviors regarding public health recommendations.

We placed emphasis on the assessment of different demographic indicators as uniquely predicting reactions to reduced mask wearing, monetary incentive programs, and work vaccination requirements (e.g., race as a predictor of reactions while adjusting for vaccination intention, age, gender, etc.). We specifically focused on comparing reports of being more likely to get vaccinated to reports of being less likely to get vaccinated; however, further analysis could highlight differences centered on the middle “no impact” response option (i.e., no impact vs. less likely and more likely vs. no impact). General intention to get vaccinated and political leaning served as meaningful and consistent predictors in all models, and in turn, they should be included in future analyses and intervention development/implementation (cf. [23]). Various other individual differences emerged that are also of note and expand our understanding of differential reactions to reduced mask-wearing and efforts to increase vaccinations. For example, when adjusting for all other demographic indicators, those with high school education or less were more likely to get vaccinated in response to a work requirement than those with more than a high school education. Similarly, those with lower household incomes were more likely to get vaccinated than those from higher income brackets. These findings indicate that work vaccination requirements might produce inequality in autonomy over one’s health decision making. As another example, White respondents reported more positive intentions to get vaccinated in response to monetary incentive programs relative to Black respondents. Future research should investigate contributing factors for this difference, including differential mistrust stemming from historic mistreatment of Black populations. Additional factors could be assessed to further this line of work, such as differences based on religion. Emphasizing individual differences in reactions to changing public health practices and deliberate efforts to increase intentions to get vaccinated are necessary, because without such emphasis, messages to encourage immunization could backfire, negatively impacting vaccine uptake.

Finally, there were substantial numbers of respondents for whom reduced mask-wearing, monetary incentive programs, and work vaccination requirements were reported to have no impact on vaccination intention. For example, 76% of those resistant to vaccination reported that changes in local masking behavior did not impact their intentions. The increased “no impact” or “neutral” response among resistant respondents is likely a reflection of the hardening of vaccination intentions among a proportion of the unvaccinated population, highlighting the difficulty that public health officials face when designing efforts to move many unvaccinated individuals into the “vaccinated column.” An alternative explanation is that researchers, public health officials, and policy makers have not yet “found” the optimal ingredients to impact intentions.

### Limitations and future directions

There are several limitations that we should note. First, our findings are limited to the Web-using population, and in turn, we were not able to hear from respondents who do not have access to the Web. However, our use of RIWI’s novel online data collection technology allowed us to reach the most diverse Web-population possible using nonprobability sampling, serving as a strength relative to other online data collection platforms (e.g., panel-based research). All Web-users in the US had a chance of encountering this survey, thus minimizing self-selection biases, and maximizing our reach to a broader set of respondents who might otherwise not participate in research. Future research could consider alternative, non-Web-based forms of data collection as a means of reaching non-Web-using respondents (e.g., random digit dialing procedures); however, those methods of course come with their own limitations.

Second, we observed a high drop off in respondents from opt-in to survey completion. Of note, RIWI’s methodology does not allow for the collection of any personally identifying information (avoiding social desirability bias), and, in turn, no incentives are provided to respondents that would encourage their continued participation beyond general interest in the survey (avoiding incentive bias). A 22% completion rate is typical compared to other RIWI surveys in the US and expected considering the survey length and its non-incentivized nature (e.g., [24]). Future research could highlight demographic differences in drop-off to better understand which populations choose to engage (and disengage) with COVID-19 related content. In the current survey, we observed similar age and gender distributions among those who opted-in to participate and those who completed the entire survey. In any case, we retained a large sample size at the conclusion of our survey that provided substantial power for the analyses.

Third, we were unable to confirm the vaccination status given by respondents, and instead had to rely on self-reported vaccination status. Among our total opt-in sample, 69.7%, 95% CI [69.4%, 70.1%] of respondents self-reported that they had received at least one dose of a COVID-19 vaccine (manuscript submitted for publication). This is comparable to the CDC’s reported adult vaccination rate of 67.9% midway through our data collection period (on July 15^th^, 2021) [25]. Although we were unable to confirm vaccination status, this comparison suggests that what people are reporting is similar to what the active vaccine coverage data shows.

We aimed to measure reactions to changing masking behavior and efforts to increase vaccination (i.e., monetary incentives and work vaccination requirements) by directly asking respondents to report on such reactions. It is possible that reactions are more nuanced than this and require more comprehensive measures for deeper understanding, which could be achieved in focus groups or key informant interviews. Our findings are also limited in providing a theoretical extension to the existing literature on reactance and public health policy. However, these limitations are offset by the strengths of producing actionable evidence that is accessible to public health policy makers and communication programs and can be immediately applied in a real-world setting.

Finally, future research might wish to assess the efforts relative to one another, and pinpoint which effort works best (or works worst) for different demographic groups. While we focused on within-effort demographic differences in this report, a comparative analysis could prove useful to those who are determining which policies to implement in their communities.

## Conclusion

The findings of this report suggest that the targeting of vaccine uptake strategies needs to be aligned with those populations who behave quite differently. For example, unvaccinated Black respondents were more likely to lean toward vaccination than unvaccinated White respondents, however White respondents were more likely than Black respondents to report positive reactions to monetary incentive programs. Rural residents not only had lower vaccination coverage and more resistance to vaccination relative to urban dwellers, but they were also more resistant to monetary incentive programs and work vaccination requirements, and in turn, may require novel, targeted messaging/incentives. Awareness of such nuances, and the ability to gauge them quickly and broadly, can assist practitioners, policy makers, and public health officials in designing and implementing communication strategies and policies that will yield optimal outcomes.

## Supporting information

S1 Fig Survey progression. (DOCX)

S1 Table Chi-Square analyses predicting anticipated reactionary response among those who would not get vaccinated in response to a work vaccination requirement by demographics. (DOCX)

S2 Table Correlations among reactions to reduced masking, monetary incentives, and work vaccination requirements. (DOCX)

S1 File Survey questions and skip/display logic. (DOCX)

## References

[1] Centers for Disease Control and Prevention. COVID data tracker [Internet]. Atlanta: US Department of Health and Human Services, CDC; 2022 Jan 5 [cited 2022 Jan 5]. Available from: https://covid.cdc.gov/covid-data-tracker

[2] Fowlkes A, Gaglani M, Groover K, Thiese MS, Tyner H, Ellingson, K, et al. Effectiveness of COVID-19 vaccines in preventing SARS-CoV-2 infection among frontline workers before and during B.1.617.2 (Delta) variant predominance—Eight U.S. locations, December 2020—August 2021. MMWR Morb Mortal Wkly Rep. 2021;70(34):1167–9. doi: 10.15585/mmwr.mm7034e4 34437521 PMC8389394

[3] Tartof SY, Slezak JM, Fischer H, Hong V, Ackerson BK, Ranasinghe ON, et al. Effectiveness of mRNA BNT162b2 COVID-19 vaccine up to 6 months in a large health system in the USA: A retrospective cohort study. Lancet. 2021;398(10309):1407–16. doi: 10.1016/S0140-6736(21)02183-8 34619098 PMC8489881

[4] Hamel L, Lopes L, Sparks G, Kirzinger A, Kearney A, Stokes M, et al. KFF COVID-19 vaccine monitor: September 2021 [Internet]. San Francisco: Kaiser Family Foundation; 2021 Sep 28 [cited 2022 Jan 5]. Available from: https://www.kff.org/coronavirus-covid-19/poll-finding/kff-covid-19-vaccine-monitor-september-2021/

[5] Roy B, LeBlanc M. COVID-19 vaccine incentives [Internet]. Washington, D.C.: National Governors Association; 2021 Aug 19 [cited 2022 Jan 5]. Available from: https://www.nga.org/center/publications/covid-19-vaccine-incentives/

[6] Messenger M. From McDonald’s to Goldman Sachs, here are the companies mandating vaccine for all or some employees. NBC News [Internet]. 2021 Aug 3 [updated 2021 Nov 16, cited 2022 Jan 5]. Available from: https://www.nbcnews.com/business/business-news/here-are-companies-mandating-vaccines-all-or-some-employees-n1275808

[7] Largent EA, Miller FG. Problems with paying people to be vaccinated against COVID-19. JAMA. 2021;325(6):534–5. doi: 10.1001/jama.2020.27121 33404585

[8] Largent EA, Persad G, Sangenito S, Glickman A, Boyle C, Emanuel EJ. US public attitudes toward COVID-19 vaccine mandates. JAMA Netw Open. 2020;3(12):e2033324. doi: 10.1001/jamanetworkopen.2020.33324 33337490 PMC7749443

[9] Schonlau M, Couper MP. Options for conducting web surveys. Stat Sci. 2017;32(2):279–92.

[10] Kim J, Bell GA, Ratcliffe HL, Moncada L, Lipsitz S, Hirschhorn LR, et al. Predictors of patient-reported quality of care in low- and middle-income countries: a four-country survey of person-centered care. Int J Qual Health Care. 2021;33(3):1–8. doi: 10.1093/intqhc/mzab110 34318883 PMC8519224

[11] Roder-DeWan S, Gage AD, Hirschhorn LR, Twum-Danso NAY, Liljestrand J, Asante-Shongwe K, et al. Expectations of healthcare quality: A cross-sectional study of internet users in 12 low- and middle-income countries. PLoS Med. 2019:16(8):e1002879. doi: 10.1371/journal.pmed.1002879 31390364 PMC6685603

[12] Roder-DeWan S, Gage A, Hirschhorn LR, Twum-Danso NAY, Liljestrand J, Asante-Shongwe K, et al. Level of confidence in and endorsement of the health system among internet users in 12 low-income and middle-income countries. BMJ Glob Health. 2020;5(8):e002205. doi: 10.1136/bmjgh-2019-002205 32859647 PMC7454186

[13] Seeman N. Use data to challenge mental health stigma. Nature. 2015;582:309. doi: 10.1038/528309a 26672520

[14] Seeman NL, Seeman MV. An anonymous survey method for obtaining sensitive, personal, or embarrassing information in the context of mental illness stigma. SAGE Research Methods Cases. 2020. [cited 2022 Jan 5]. Available from: 10.4135/9781529719260

[15] Seeman N, Tang S, Brown AD, Ing A. World survey of mental illness stigma. J Affect Disord. 2016;190:115–121. doi: 10.1016/j.jad.2015.10.011 26496017

[16] Seeman N, Ing A, Rizo C. Assessing and responding in real time to online anti-vaccine sentiment during a flu pandemic. Healthc Q. 2010;13:8–15. doi: 10.12927/hcq.2010.21923 20959725

[17] Khubchandani J, Sharma S, Price JH, Wiblishauser MJ, Sharma M, Webb FJ. COVID-19 vaccination hesitancy in the United States: A rapid national assessment. J Community Health. 2021;46:270–7. doi: 10.1007/s10900-020-00958-x 33389421 PMC7778842

[18] Nguyen LH, Joshi AD, Drew DA, Merino J, Ma W, Lo C, et al. (2021). Racial and ethnic differences in COVID-19 vaccine hesitancy and uptake. medRxiv [Preprint]. 2021. [cited 2022 Jan 5]. Available from: doi: 10.1101/2021.02.25.21252402 33655271 PMC7924296

[19] Phillips N. The coronavirus is here to stay—here’s what that means. Nature. 2021;590, 382–4. doi: 10.1038/d41586-021-00396-2 33594289

[20] Sprengholz P, Eitze S, Felgendreff L, Korn L, Betsch C. Money is not everything: Experimental evidence that payments do not increase willingness to be vaccinated against COVID-19. J Med Ethics. 2021;47(8):547–8. doi: 10.1136/medethics-2020-107122 33602717

[21] Abusaid S. DeKalb vaccine drive draws more than 2,500 to Stonecrest mall. The Atlanta Journal-Constitution [Internet]. 2021 Aug 28 [cited 2022 Jan 5]. Available from: https://www.ajc.com/news/dekalb-vaccine-drive-draws-more-than-2500-to-stonecrest-mall/OP7D63YKRVBJJGA2EJKXP5FY3Q/

[22] Cooper H, Steinhauer J. The U.S. military will mandate Covid-19 vaccines for troops. The New York Times [Internet]. 2021 Aug 9 [updated 2021 Nov 2, cited 2022 Jan 5]. Available from: https://www.nytimes.com/2021/08/09/us/politics/vaccine-mandate-military-covid.html

[23] Siegler AJ, Luisi N, Hall EW, Bradley H, Sanchez T, Lopman BA, et al. Trajectory of COVID-19 vaccine hesitancy over time and association of initial vaccine hesitancy with subsequent vaccination. JAMA Netw Open. 2021;4(9):e2126882. doi: 10.1001/jamanetworkopen.2021.26882 34559232 PMC8463937

[24] Elevyst, RIWI Corp., PRO-A. Addressing drug use and recovery stigma: Initial findings released [Internet]. Toronto: RIWI Corp.; 2021 Aug 24 [cited 2022 Jan 5]. Available from: https://riwi.com/research/addressing-drug-use-recovery-stigma-initial-findings-released/

[25] Centers for Disease Control and Prevention. COVID-19 vaccinations in the United States, jurisdiction [dataset]. 2022 Jan 4 [cited 2022 Jan 4]. Available from: https://data.cdc.gov/Vaccinations/COVID-19-Vaccinations-in-the-United-States-Jurisdi/unsk-b7fc

