# Supplementary material for: Masks, money, and mandates: A national survey on efforts to increase COVID-19 vaccination intentions in the United States
Source: PLoS One. 2022 Apr 21;17(4):e0267154. doi: 10.1371/journal.pone.0267154 (PMC9022841; doi:10.1371/journal.pone.0267154)
Supplement: S1 Fig — (DOCX) [file pone.0267154.s001.docx]

Survey Start Date:

June 30^th^, 2021

Exposed *N*:

1,026,850

Vaccinated *N*:

44,524

Complete *N*:

10,866

Survey Close Date:

July 26^th^, 2021

Complete *N*:

14,152

Opt-in *N*:

63,853

Unvaccinated *N*:

19,329

Complete *N*:

3,286

**S1 Fig. Survey progression from initiation to completion regarding general survey participation.** Opt-in *N* refers to respondents who were eligible to participate (18+ years old, located in the US) and who answered questions assessing age, gender, and vaccination status.
