# Supplementary material for: Masks, money, and mandates: A national survey on efforts to increase COVID-19 vaccination intentions in the United States
Source: PLoS One. 2022 Apr 21;17(4):e0267154. doi: 10.1371/journal.pone.0267154 (PMC9022841; doi:10.1371/journal.pone.0267154)
Supplement: S1 Table — (DOCX) [file pone.0267154.s002.docx]

**S1 Table. Chi-Square analyses predicting anticipated reactionary response among those who would not get vaccinated in response to a work vaccination requirement by demographics.**

|  | **Quit Job** | **Protest** | **Legal Action** | **Other** | ***p*** |
| --- | --- | --- | --- | --- | --- |
| **Vaccination Intention** |  |  |  |  |  |
| Resistant | 241 (30.2%) | 62 (7.8%) | 354 (44.4%) | 141 (17.7%) | .002 |
| Leaning toward | 30 (29.1%) | 19 (18.4%) | 34 (33.0%) | 20 (19.4%) |  |
| **Age** |  |  |  |  |  |
| 18-25 | 69 (36.1%) | 26 (13.6%) | 53 (27.7%) | 43 (22.5%) | .004 |
| 26-35 | 52 (28.0%) | 16 (8.6%) | 83 (44.6%) | 35 (18.8%) |  |
| 36-45 | 46 (27.5%) | 15 (9.0%) | 77 (46.1%) | 29 (17.4%) |  |
| 46-55 | 46 (30.3%) | 12 (7.9%) | 69 (45.4%) | 25 (16.4%) |  |
| 56-64 | 25 (24.3%) | 3 (2.9%) | 63 (61.2%) | 12 (11.7%) |  |
| 65-74 | 13 (29.5%) | 4 (9.1%) | 22 (50.0%) | 5 (11.4%) |  |
| 75-84 | 3 (50.0%) | 0 (—%) | 1 (16.7%) | 2 (33.3%) |  |
| 85+ | 17 (32.7%) | 5 (9.6%) | 20 (38.5%) | 10 (19.2%) |  |
| **Gender** |  |  |  |  |  |
| Male | 128 (28.0%) | 48 (10.5%) | 208 (45.5%) | 73 (16.0%) | .023 |
| Female | 124 (33.1%) | 22 (5.9%) | 152 (40.5%) | 77 (20.5%) |  |
| Other | 19 (27.5%) | 11 (15.9%) | 28 (40.6%) | 11 (15.9%) |  |
| **Race** |  |  |  |  |  |
| White | 168 (31.5%) | 43 (8.1%) | 254 (47.6%) | 69 (12.9%) | <.001 |
| Black | 36 (33.0%) | 8 (7.3%) | 29 (26.6%) | 36 (33.0%) |  |
| Latinx | 20 (26.0%) | 9 (11.7%) | 28 (36.4%) | 20 (26.0%) |  |
| Asian | 3 (20.0%) | 5 (33.3%) | 4 (26.7%) | 3 (20.0%) |  |
| NA/AN | 10 (31.3%) | 4 (12.5%) | 15 (46.9%) | 3 (9.4%) |  |
| Other | 21 (26.6%) | 6 (7.6%) | 36 (45.6%) | 16 (20.3%) |  |
| Multi-racial | 13 (23.6%) | 6 (10.9%) | 22 (40.0%) | 14 (25.5%) |  |
| **Education** |  |  |  |  |  |
| High school or less | 102 (31.7%) | 32 (9.9%) | 114 (35.4%) | 74 (23.0%) | .023 |
| Tech/vocational training | 45 (29.8%) | 10 (6.6%) | 73 (48.3%) | 23 (15.2%) |  |
| College degree | 79 (28.8%) | 29 (10.6%) | 130 (47.4%) | 36 (13.1%) |  |
| Masters or higher | 45 (29.2%) | 10 (6.5%) | 71 (46.1%) | 28 (18.2%) |  |
| **Living Location** |  |  |  |  |  |
| Large city | 70 (31.5%) | 15 (6.8%) | 91 (41.0%) | 46 (20.7%) | .310 |
| Suburb | 65 (27.1%) | 25 (10.4%) | 117 (48.8%) | 33 (13.8%) |  |
| Town/village | 68 (31.5%) | 17 (7.9%) | 87 (40.3%) | 44 (20.4%) |  |
| Rural area/farm | 68 (30.5%) | 24 (10.8%) | 93 (41.7%) | 38 (17.0%) |  |
| **Political Leaning** |  |  |  |  |  |
| Democrat | 26 (26.8%) | 8 (8.2%) | 37 (38.1%) | 26 (26.8%) | .029 |
| Independent, lean Democrat | 17 (38.6%) | 8 (18.2%) | 12 (27.3%) | 7 (15.9%) |  |
| Independent | 98 (31.2%) | 28 (8.9%) | 125 (39.8%) | 63 (20.1%) |  |
| Independent, lean Republican | 43 (27.0%) | 14 (8.8%) | 83 (52.2%) | 19 (12.0%) |  |
| Republican | 87 (30.3%) | 23 (8.0%) | 131 (45.6%) | 46 (16.0%) |  |
| **Income** |  |  |  |  |  |
| Under $20,000 | 67 (35.4%) | 21 (11.1%) | 55 (29.1%) | 46 (24.3%) | <.001 |
| $20,000 - $50,000 | 67 (31.3%) | 23 (10.7%) | 81 (37.9%) | 43 (20.1%) |  |
| $50,001 - $75,000 | 39 (25.0%) | 13 (8.3%) | 78 (50.0%) | 26 (16.7%) |  |
| $75,001 - $125,000 | 38 (27.0%) | 5 (3.5%) | 86 (61.0%) | 12 (8.5%) |  |
| $125,001 - $250,000 | 29 (32.2%) | 8 (8.9%) | 43 (47.8%) | 10 (11.1%) |  |
| Over $250,000 | 31 (27.9%) | 11 (9.9%) | 45 (40.5%) | 24 (21.6%) |  |

P-value obtained from Chi-square test. Row (vs. column) percentages displayed. All chi-square tests conducted independently, without adjusting for other variables. Income = annual household income.
