# Supplementary material for: Masks, money, and mandates: A national survey on efforts to increase COVID-19 vaccination intentions in the United States
Source: PLoS One. 2022 Apr 21;17(4):e0267154. doi: 10.1371/journal.pone.0267154 (PMC9022841; doi:10.1371/journal.pone.0267154)
Supplement: S2 Table — (DOCX) [file pone.0267154.s003.docx]

**S2 Table. Correlations among reactions to reduced masking, monetary incentives, and work vaccination requirements.**

|  | 1 | 2 | 3 | 4 |
| --- | --- | --- | --- | --- |
| 1. Reduced Masking |  |  |  |  |
| 1. Gift card | .27 [.23, .30] |  |  |  |
| 1. Lottery | .24 [.21, .27] | .70 [.68, .71] |  |  |
| 1. Work requirement | .31 [.27, .35] | .41 [.38, .45] | .43 [.40, .47] |  |
| *M* (*SD*) | 1.04 (0.61) | 0.86 (0.67) | 0.90 (0.69) | 0.90 (0.86) |

All correlation coefficients significant at *p*  < .001. 95% confidence intervals displayed in brackets.
