# Supplementary material for: Masks, money, and mandates: A national survey on efforts to increase COVID-19 vaccination intentions in the United States
Source: PLoS One. 2022 Apr 21;17(4):e0267154. doi: 10.1371/journal.pone.0267154 (PMC9022841; doi:10.1371/journal.pone.0267154)
Supplement: S1 File — (DOCX) [file pone.0267154.s004.docx]

**S1 File. Survey questions and skip/display logic.**

*Note. Skip/display logic specified in italics*

What is your age?

What is your gender?

Male

Female

Other

*For those who chose “other”*

You indicated "other" for your gender. Please provide more details on your gender identity in the box below or press the button to skip ahead.

[open]

*For everyone*

Have you personally received the COVID-19 vaccine?

Yes, a single-dose vaccine (J&J)

Yes, the first of two doses (Moderna or Pfizer)

Yes, both doses of a two-dose vaccine (Moderna or Pfizer)

I have not received a vaccination

*For those vaccinated*

Did you have the choice to get a different type of vaccine?

Yes, I had the choice to get a single-dose vaccine (J&J)

Yes, I had the choice to get a two-dose vaccine (Pfizer or Moderna)

No, I did not have the choice to get a different type of vaccine

What month did you receive your most recent COVID-19 vaccine dose?

December

January

February

March

April

May

June

July

I am not sure

*For those vaccinated with J&J in April*

Did you receive your vaccine before or after the Johnson & Johnson distribution pause? Distribution was paused on April 13th, 2021 and restarted April 25th, 2021

Before - April 13th or earlier

After - April 25th or later

*For those vaccinated with J&J after April 25th*

Use of the J&J vaccine was paused on April 13th to investigate reports of very rare and severe blood clots. Vaccine use was then reinstated April 25th after the FDA and CDC performed a safety review and found that the risk was very low and the vaccine benefits outweigh the risks. When you were vaccinated, was any information about this safety issue with the J&J vaccine provided?

Yes, someone discussed it with me, and I received written materials describing it

Yes, someone discussed it with me, but I did not receive any written materials describing it

No one discussed it with me, but I did receive a fact sheet that mentioned it

No one discussed it with me, and I did not receive any materials mentioning it

*For everyone*

How do you identify? Select all that apply

White

African American or Black

Hispanic / LatinX

Asian

Native American or American Indian

Alaskan Native

Other ethnic group

No more apply, continue

*For those who chose “other”*

You chose "other" in the previous question. How do you identify?

[open]

*For those unvaccinated*

Do you plan to get the COVID-19 vaccine?

I will definitely get it as soon as I can

I will likely get it as soon as I can

I will likely get it but not right away

I will likely not get vaccinated

I will definitely not get vaccinated

Have you had COVID-19?

Yes

No

Unsure

How often do you currently wear a mask in stores?

Never

Only if the store requires me to

Sometimes

Always (or almost always)

Since the new policy guidelines on mask wearing, fewer people are wearing masks in public settings. How does that shift make you feel?

Very concerned

Moderately concerned

A little concerned

Not at all concerned

I wasn't aware of the shift

Are you more or less likely to get a COVID-19 vaccine now that fewer people are wearing masks in public?

More likely

Less likely

No effect

If I was given an incentive like a $25 gift card for getting fully vaccinated against COVID-19...

I would be more likely to get vaccinated right away

I would be less likely to seek vaccination

There would be no effect on my getting vaccinated

If I was automatically enrolled in a lottery when I got fully vaccinated against COVID-19 and could win $100,000...

I would be more likely to get vaccinated right away

I would be less likely to seek vaccination

There would be no effect on my getting vaccinated

If my employer required me to get the COVID-19 vaccine...

I would get vaccinated

I would not get vaccinated

I am not sure what I would do

I am self-employed

I am unemployed

I am retired

I am a stay-at-home parent and/or homemaker

*For those who would not get vaccinated*

You said you would not get vaccinated if your employer required it. What would you do instead?

Quit my job

Protest

Consider legal action

Other

*For those unvaccinated who would not get it right away*

What are your main reasons against getting a COVID-19 vaccine? Select up to 4

The medical system has mistreated people like me

People like me were not part of the vaccine studies

I am not at risk of getting sick from COVID-19

The vaccines will not protect me

I am worried about vaccine safety

I am worried about feeling ill from the vaccine

It is inconvenient to get the vaccine

I have a health condition and do not know how the vaccine will impact me

Herd immunity (enough people are vaccinated that I don't need it)

It is against my religion

The vaccine information is confusing

The pandemic is almost over anyway

The vaccine development was rushed

I don't like needles/injections

Other reason

No more apply, continue

*For those chose “other”*

What is your other reason for not wanting to get the vaccine?

[open]

*For those who selected more than one reason to not get vaccinated (not including “other”)*

Of the vaccine concerns you selected, what is your most important concern?

[list of their selected concerns]

*For those unvaccinated*

Have you discussed getting the COVID-19 vaccine with your doctor or other health care provider?

Yes

No

*For those who said “yes”*

What did your doctor or other health care provider tell you?

My doctor encouraged me to get the vaccine

My doctor encouraged me to not get the vaccine

I didn't understand my doctor's recommendation

My doctor would not tell me whether or not I should get the vaccine

*For those who said “no”*

Why have you not discussed the COVID-19 vaccine with your doctor or another health care provider?

I don’t have a doctor or other health care provider

I have not seen my doctor or other health care provider

My doctor or health care provider did not want to talk to me about the vaccine

I don’t need to talk about this with my doctor or other health care provider. I can make my own decisions

*For everyone*

Are you aware of any recent reports (within the last couple of weeks) of side effects associated with COVID-19 vaccines?

Yes

No

*For those who said “yes”*

Which part of the body was reported to be affected by the COVID-19 vaccines? (Select all that apply)

Brain

Lung

Skin

Blood

Heart

Liver

Bone

None of the above

No more, continue

*For those unvaccinated who chose “heart”*

Does this information make you:

More likely to get vaccine

No impact on whether I would get vaccine

Less likely to get vaccine

*For everyone*

Where do you primarily get the news of the day that’s important to you?

Social media (like Reddit, Facebook, Twitter, Instagram, TikTok)

Television network news

Television local news

Late night talk shows

Hard copy newspaper

Online newspaper

Radio

Podcast

Other

*For those who chose “other”*

Where else do you get the news of the day from?

[open]

*For those who chose “television network news”*

Which network news stations do you usually watch? (Select all that apply)

FOXNEWS

CNN

CBS

ABC

NBC

MSNBC

CNBC

Other

No more apply, continue

*For those who chose “other”*

What other network news stations do you watch?

[open]

*For those who chose “hard copy newspaper”*

Which hard copy newspapers do you usually read? (Select all that apply)

USA Today

The Wall Street Journal

The Washington Post

The New York Times

My local newspaper

Other

No more apply, continue

*For those who chose “online newspaper”*

Which online newspapers do you usually read? (Select all that apply)

USA Today

The Wall Street Journal

The Washington Post

The New York Times

Other

No more apply, continue

*For those who chose “other”*

What other online newspapers do you read?

[open]

*For those who chose “podcasts”*

Which podcasts do you usually listen to for news updates? (Select all that apply)

The Daily

Up First

NPR News Now

Pod Save America

The Globalist

World Business Report

The Journal

What a Day

Other

No more apply, continue

*For those who chose “other”*

What other podcasts do you usually listen to?

[open]

*For everyone*

For entertainment, I... (select all that apply)

Watch cable TV (HGTV, BET, etc.)

Use social media (Instagram, Twitter, Tiktok, Facebook, etc.)

Watch shows or movies on a streaming service (Netflix, Hulu, Amazon, Disney+, etc.)

Read a book

Stream music on a service like Spotify, Apple Music, Pandora or similar

Listen to the radio

Other

No more apply, continue

*For those who chose “other”*

Where else do you go for entertainment?

[open]

*For everyone*

What is the highest level of education you have completed?

High School degree or less

Technical or vocational training

College Degree

Masters degree or higher

How would you describe the area in which you live?

Large city

Suburb

Town / village

Rural area / farm

In general, do you think of yourself politically as…

Democrat

Independent but I lean Democrat

Independent

Independent but I lean Republican

Republican

Approximately how much is your annual household income? (Before taxes)

Under $20,000

$20,000 - $50,000

$50,001 - $75,000

$75,001 - $125,000

$125,001 - $250,000

Over $250,000
